# Mental Mechanisms for Topics Identification

**DOI:** 10.1155/2014/920892

**Published:** 2014-03-13

**Authors:** Louis Massey

**Affiliations:** Department of Mathematics and Computer Science, Royal Military College, Kingston, ON, Canada K7K 7B4

## Abstract

Topics identification (TI) is the process that consists in determining the main themes present in natural language documents. The current TI modeling paradigm aims at acquiring semantic information from statistic properties of large text datasets. We investigate the mental mechanisms responsible for the identification of topics in a single document given existing knowledge. Our main hypothesis is that topics are the result of accumulated neural activation of loosely organized information stored in long-term memory (LTM). We experimentally tested our hypothesis with a computational model that simulates LTM activation. The model assumes activation decay as an unavoidable phenomenon originating from the bioelectric nature of neural systems. Since decay should negatively affect the quality of topics, the model predicts the presence of short-term memory (STM) to keep the focus of attention on a few words, with the expected outcome of restoring quality to a baseline level. Our experiments measured topics quality of over 300 documents with various decay rates and STM capacity. Our results showed that accumulated activation of loosely organized information was an effective mental computational commodity to identify topics. It was furthermore confirmed that rapid decay is detrimental to topics quality but that limited capacity STM restores quality to a baseline level, even exceeding it slightly.

## 1. Introduction

The topics of a natural language text are the main themes it discusses. Topics are useful because they convey the high level, general meaning of a text. Even though topics are a very basic form of meaning, they provide valuable information that can be exploited during further semantic and syntactic analysis. Topics can also be used directly in a variety of information management tasks. For instance, one may want to identify topics to decide if a text is of interest during an information search or literature review.

Topics identification (TI) is the process by which topics are determined. TI has been modeled computationally in various ways such as Latent Dirichlet Allocation [1], Latent Semantic Analysis [2, 3], text clustering [4, 5], document classification [6, 7], and keyword extraction [8–10]. These approaches have been highly successful, and our aim is not to propose efficiency or effectiveness evolutions. We are rather considering the following question: what are the brain mechanisms involved in identifying topics? This is an interesting question because understanding how the brain derives meaning, even in the basic form of topics, can lead to improved understanding of natural intelligence and inspiration for new artificial intelligence techniques.

The current psycholinguistic TI paradigm sets as a basic requirement that learning from linguistic data must be part of the cognitive process of determining meaning [11–14]. We take a divide and conquer approach and suggest that the mechanisms involved in TI could be better understood if viewed separately from learning. Freed from the presence of a training dataset, we put the emphasis on how TI takes place for single documents. This is what a person might do when reading a document for which he or she already possesses the necessary knowledge; that is, the person does not need to acquire new information to make sense of the text presently perused. This view allows for the investigation of TI mental mechanisms exploiting previously acquired knowledge. We particularly suggest that topics are determined by frequently recurring* ideas* as a person reads. This is another difference with most existing TI models that rely on a geometric approach with high-dimensional spatial representation and linear algebraic computations. Our main question then becomes as follows: what are the mental mechanisms involved in computing the frequency of recurring ideas?

A second question that arises as a consequence of the previous one is as follows: what is the nature of these so-called ideas? Since the early days of artificial intelligence research, it has been assumed that intelligent systems had to rely on structured knowledge stored, for example, in semantic networks or as logical rules. In implementing artificial systems and computational models to test theories, one faced a “knowledge acquisition bottleneck” due to the intensity of the work required to handcraft knowledge [15, 16]. We suggest that formal knowledge structures are not needed and that “loosely” organized mental information—what we call ideas—suffices to identify topics. This work investigates how this form of information is exploited to derive topics. Some may contest that merely exploiting existing knowledge, whatever its form, avoids the hardest and most interesting problem of knowledge acquisition. We do not deny the importance and difficulty of learning and representing knowledge: we reiterate that our aim is a more focused investigation of the TI process itself by isolating it from learning. In other words, we distinguish between two related but separate processes: one is learning and encoding information about words (their “meanings”), and the other is a mechanism that constructs the overall “meaning” of a document (its topics) from existing word meaning information. We focus on the latter.

Our main hypothesis is that topics are the result of accumulated neural activation of loosely organized ideas encoded in long-term memory (LTM) neural networks. We claim that this is the basic brain mechanism involved in computing the frequency of recurring ideas, thus answering our primary question stated previously. We experimentally tested this hypothesis with a computational model that simulates LTM activation. The model furthermore posits that activation must necessarily decay due to its bioelectrical nature. A resulting second hypothesis is that decay will negatively affect topics identification since it should result in loss of information during processing. The model also predicts the presence of short-term memory (STM) that keeps the focus of attention on a small set of words, thus temporarily delaying decay of their associated LTM ideas. Our third hypothesis is that limited capacity STM should counteract the negative effect of decay. Our results confirmed all three hypotheses as correct.

The paper will start by describing the computational model used in our experiments and explaining its main underlying principles. We then follow with a section on our experimental methodology, and finally we present and discuss results.

## 2. The ReAD Model

ReAD is a model of how the brain processes information when someone reads and identifies topics in natural language text. The model assumes that individual words have previously been recognized. ReAD stands for REtrieval Activation and Decay, corresponding to the actions taking place for each word read from the text. More specifically, these actions involve the following: (1) reading a word triggers the retrieval of information stored in LTM, which physically takes the form of neural activation [17, 18]; and (2) LTM activation decays over time when a word is not under the focus of attention. LTM memories themselves do not decay: it is the activation associated with their retrieval that does. The process is unconscious other than the final realization of what the topics are. The topics are derived from the accumulation of activation caused by recurrent retrieval of the same LTM information. We suggest that this process might be innate: although we learn to read (i.e., to recognize letters and words, to construct a detailed mental understanding of the text), the fundamental mental mechanisms to unconsciously activate information associated with words and to identify topics based on accumulated decaying activation could very well be naturally present in the brain. This process may as well form a basis for proto-understanding not only of textual inputs but also of other stimuli such as language audition and visual recognition. This is conjecture at the moment, but worthy of further investigation.

LTM information corresponds to “ideas”—the knowledge that someone has accumulated about words—coded in brain neural networks. As explained in the introduction, how ideas were acquired and how exactly they are encoded in neural networks are not relevant for the purpose of this work. The important point is that, given a word as stimulus, the ideas related to that word can be retrieved, causing the neural networks storing the information to become activated. Ideas are thus information embodied in neural networks about what words “mean” to a reader. The meaning of words is generally polysemous and includes both connotative and denotative senses acquired from personal experience, education, and cultural background. The ReAD model posits that ideas are “loosely” organized, in that no formal structure is present. This is another important difference between this work and existing literature that often theorizes formal semantic structures. For instance, with the ReAD model the word “red” may cause the retrieval of diverse ideas such as “blood,” “love,” “color,” “stop sign,” and “danger.” One can visualize the association between the word “red” and its multiple ideas as simple neural pathways connecting the word “red” to each of these ideas, causing them to become activated when one reads or mentally focuses on that word. There is no other structure or information explicitly stating semantic relationships such as “red* is a* color” or a “stop sign* has property* color* set to value* red.”

ReAD does not perform word sense disambiguation and implements exhaustive access; that is, all senses of polysemous words are equally activated [19, 20]. Appropriate word sense is determined indirectly over time as a side effect of the TI process. Considering the example above with word “red,” all ideas associated with it would be equally activated. Assuming a text that discusses the theme of love, previous and subsequent words in the text would also likely activate the idea “love.” Over time, idea “love,” the correct sense intended for word “red,” would accumulate more activation. It is important to realize that the aim is not to disambiguate specific word senses at the moment a word is read but rather to find the topics of the whole text.

Hence, as more words are read, more ideas become activated, with some words further activating the same ideas. The final result is higher activation of a small set of ideas, effectively pointing out the main ideas—the topics—of the text. Basically, ReAD discovers ideas that occur frequently, using activation as the computational commodity to determine frequency. This is the fundamental tenet behind the ReAD model. To summarize in more conventional terms, the “overall meaning” of a text, expressed by its topics, is a composition of the individual meanings of each word, with semantically related words progressively constructing meaning [21]. Semantic relationships between words are not expressed formally; they are elicited by activating LTM neural networks storing common ideas.

There is also a temporal aspect to take into account because activation is not permanent. Indeed, due to its bioelectric nature, neural activation in LTM must necessarily decay with time. ReAD assumes this to be an unavoidable behavior. This could be problematic because of the loss of information as one proceeds through a text. To counteract the detrimental effect that decay may have on topics identification, ReAD includes short-term memory (STM) to store a few recently fixated words. This causes the unconscious upkeep of the focus of attention on these words, thus temporarily avoiding decay of the associated ideas in LTM. Since STM has limited capacity [22], it is subjected to interference. When it has reached maximum capacity and a new word is read, the word that has been in STM for the longest time without rehearsal is replaced by the new word. When a word is not in STM anymore, the focus of attention on the associated neural networks in LTM is lost, and decay is initiated. Decay is thus controlled by two parameters: first, STM capacity determines how long decay is delayed, and, second, the rate of decay determines how rapidly neural networks return to their resting value.

The ReAD model posits that activation and decay of LTM ideas coupled with limited capacity STM are the fundamental mental mechanisms of TI. With decaying activation as computational commodity, the brain identifies topics by calculatingthe frequency of ideas as words from a text are read. we anticipate that this process could equally apply to verbal natural language. Once reading is completed, which could be at the actual end of a text or at any point within it, one is able to consciously realize what they “have on their mind,” to use a common expression. This essentially consists in accessing the ideas stored in highly activated neural networks. The higher the activation is, the more facilitated and prioritized the access is. From that viewpoint, the final activation serves as an estimate of a probability or evidence that the idea coded in a LTM neural network corresponds to the thematic content of a text.

One may point out that ReAD is similar to spreading activation [23, 24] but there are key differences. First, instead of using formally structured knowledge in a semantic network or other similar arrangements, as explained previously, ReAD uses loosely organized ideas. Second, the ReAD model includes neither actual spreading of activation nor decay based on distance from a main concept, instead directly retrieving and activating ideas, with decay being a temporal phenomenon.

Finally, we emphasize that ReAD does not aim at modeling the specific workings of neural networks involved in natural language understanding [25, 26]. Also, ReAD provides neither a detailed cognitive architecture nor a neuroanatomical model of language understanding [27] but rather aims at outlining the general mental process involved in TI.

## 3. Materials and Methods

All data necessary to reproduce our experiments are available at http://www.louismassey.com/papers/dataExpDecay-STM.rar.

### 3.1. ReAD Implementation and Dictionary

We implemented ReAD in computer software according to Algorithm 1. In order to simulate the loosely organized knowledge stored in someone's LTM, we relied on an electronic dictionary. For our purpose, there are two main problems with dictionaries: they do not capture the full wealth of information present in the human brain and they are somewhat structured information. To ensure a dictionary is an appropriate strategy to convey our assumptions that formally structured knowledge is not necessary in TI, we extract individual words from all definitions of a word. We thus end up with general purpose unstructured lexical information. To distinguish words obtained from the dictionary from the words in the text, we call words from the dictionary* items*. Items thus become surrogates for LTM ideas. The syntactic structure of the information obtained from the dictionary is ignored: only words, as individual items, are used. Stop words (prepositions, articles, etc.) from the text and from dictionary items are ignored because, without syntactic processing, they provide no useful information for the identification of topics. We also ignore n-grams, entities made of multiple words such as “United States of America” to keep ReAD in its most basic form where the composition of individual words constructs meaning.

WordNet 2.1 [28] is the dictionary employed in the experiments. WordNet is a lexical database organized by synonym sets (synsets) and providing a variety of relationships between words such as hypernymy. Hypernyms are parent synsets in the WordNet hierarchy. For example, the hypernym for “dog” is “canine, canid.” For our experiments, we queried WordNet for nouns only and retrieved synsets, definitions, hypernyms, and “glosses” (example usage sentences). This corresponds to options -synsn -g when calling WordNet. The assumption behind querying only for nouns is that nouns carry most of the meaning relevant to topics by identifying the concepts and objects discussed in the text.

A few points are important to note for reproducibility of our experiments and to clarify the validity of our experimental methodology: first, the items are the words from all senses with no attempt to perform disambiguation. Second, we do not perform part-of-text tagging and we query each nonstop word from the text with WordNet. If the word has a noun entry in WordNet, the information is used even though the word itself may not be used as a noun in the text. The rationale for not performing word sense disambiguation and part-of-speech tagging is to avoid any linguistic preprocessing, with the dual objective of observing ReAD's performance in its most basic form and seeing if the ReAD process itself can weed out irrelevant items. Third, we intentionally did not exploit any of the formal structure of WordNet to ensure our experiments fit within the general philosophy behind ReAD, namely, that formally organized knowledge is not required in TI. One may suggest that, by retrieving hypernyms and glosses, structured information is exploited. However, our goal is to model the wealth of information that may be activated in a person's brain, so this additional information appears to be more realistic. This addresses the second problem with dictionaries stated previously. Since we use only individual words obtained from WordNet (the items) without syntactic processing and without actually exploiting WordNet's semantic relationships, we think that our method is as consistent as possible with the notion of loosely organized LTM information.

To illustrate how the list of items is generated for a particular word from the text, we show a partial output from WordNet when querying for word “gold.” Under each numbered sense, the synset is shown with a short definition. For example, for sense 1, the synset is “gold” and its definition “coins made of gold” is shown in parenthesis. The direct hypernym for this synset “precious metal” follows in the next line, preceded by “=>”. Glosses are listed between quotations marks following a definition:

 
Sense 1
 
gold – (coins made of gold)
 
=> precious metal – (less common and valuable metals often used to make coins or jewelry)
 
Sense 2
 
amber, gold – (a deep yellow color; **“** an amber light illuminated the room **”** )
 
=> yellow, yellowness – (yellow color or pigment)

The list of items in this case would be all unique words in all senses, less stop words, namely,  gold, coins, precious, metal, common, valuable, metals, jewelry, amber, deep, yellow, color, amber, light, illuminated,  room, yellowness, pigment. Note that no linguistic processing, including no stemming or other form of morphological standardization, is performed on items, again to keep ReAD in its most basic form. Other than stop words, some undesirable words are also removed from the list of items; they are words that are too general or used too frequently in definitions to be good topics, such as thing, person, time, agency, cause, and object. In the example above, used, made, and  make were not selected as items for that reason. These undesirable words were collected from trials and errors during previous experiments and from WordNet's higher-level classes. The list is available online. We are aware that removing items in this manner is far from ideal, but it was necessary for practical reasons because these items masked useful experimental results. We think this is rather an implementation issue than a problem with the ReAD model itself, but we will nevertheless look into it in future work (see the results and discussion section for more details on this issue). We emphasize that there are a lot of other irrelevant items (e.g., in the example above:  room, common), and no attempt has been made to remove those. We only removed highly general items that kept showing up for many documents.

With respect to step (2.5.1) of Algorithm 1, decay could have been implemented in many different ways. One that may come to mind naturally is along the line of the biological neuron exponential decay. However, we are not dealing with single neuron decay here but with networks that may have overlaid decay effects, and our aim is not to investigate the particular behaviors of neurons. Additionally, our previous experiments have shown a linear decay yielded better topics. One may also observe that new items retrieved for the last words of a document cannot decay and may therefore be advantaged. Decay thus appears biased to favor items retrieved later in the document. This may be similar for human readers who might be left with a stronger impression of the last words they have read. At the same time, new items for the last words of the text do not have much time to accrue activation either, so we do not think this is a major issue.

Finally, for the output we chose the top 10 activated items as topics, as explained later in the quality evaluation section.

### 3.2. Experimental Procedure and Document Sets

Our aim is not to improve the efficiency and effectiveness of existing topics identification methods, and as such our experiments do not include a comparison between ReAD and other TI methods. Our experimental objectives match the questions and hypothesis stated previously, namely, first, to determine if activation of LTM ideas is an effective computational commodity to find highly frequent ideas and if these indeed correspond to topics of the text; and, second, to observe the effects of STM and decay on the topics found with ReAD. To achieve these objectives, we measure the quality of topics obtained with ReAD under various conditions and compare them to a baseline quality. Specifically, we ran the computational implementation of ReAD presented previously on two sets of documents while varying STM capacity and decay rate, respectively, from 0 to 10 and 0 to 25. The upper boundaries of 10 and 25 were determined from previous experiments that showed that quality tends to change little beyond these values.

The overall experimental procedure we applied to each document set is as follows:

1. For each pair STM capacity-decay rate:
  (1.1) For each document:
    (1.1) Process document with ReAD
    (1.2) Collect the top 10 activated items as topics
    (1.3) Measure topics quality (see Section 3.3 for details)
  (1.2) Sum and display topics quality of all documents

No decay (decay = 0) and no STM (STM = 0) are the control baseline. This is justified as follows. As mentioned previously, decay is assumed to be an unavoidable effect in bioelectrical neural systems that should result in lower quality. By measuring the quality of topics* without* decay, we should then obtain an ideal case that can serve as an upper quality baseline. From a nonneural perspective, meaning* without* decay, frequency of ideas is straightforward to compute with dictionary items serving as surrogates for ideas:

 
For each non-stop word in a document
   
Extract items from the word definitions in a dictionary
   
Count all non-stop and non-undesirable items
 
Output the items with the highest frequency as topics

This process is equivalent to the ReAD algorithm (Algorithm 1) without decay and STM. Based on our proposal that TI mental processing corresponds to computing ideas frequency, our goal is to observe if the topics obtained under decaying conditions can result in the same quality as without decay. By summing the quality of the top 10 frequency items of all documents in the no decay case, we obtain an upper quality baseline.

We suggested the presence of STM to counteract the negative effect of decay. Therefore, decaying conditions without STM (STM = 0) should provide an important observation of lower quality. The hypothesis we aim at verifying is that as STM capacity is increased under rapid decaying conditions, topics quality should increase from the lower quality towards the higher quality baseline (decay = 0). This would demonstrate that decaying neural activation coupled with STM can serve as mental computational commodities to estimate ideas frequencies and consequently to identify topics.

The two sets of documents used in our experiments are the Reuter-21578 Distribution 1.0 and “About pages” of various organization and company web sites. In the first case, a random subset of 246 documents was extracted from the Reuter collection. This text collection consists of news feeds in the economic domain. The second set of documents was collected by the author and comprises 72 documents falling under the following general areas: transportation, information technology, energy, pharmaceutical, food, vegetarianism/animal rights, sports, climate change, and universities. This second document set was prepared to provide a larger variety of documents compared to the specialized nature of Reuter documents. Documents were not subjected to any other preprocessing than stop word removal and stemming performed automatically by WordNet.

### 3.3. Quality Evaluation

To compute quality, we compared topics generated by ReAD with those of a “gold standard,” which are topics taken as correct because they were obtained from humans. The gold standard topics of the Reuter documents are composed of Reuter's experts solution that is included with the dataset. We replaced abbreviations in the original Reuter's experts topics by the actual words. For example, where the Reuter experts listed “nat-gas” as topic, we replaced it with “natural gas.” This was done because acronyms present in the experts topics are generally not present in Wordnet and therefore cannot be generated as topics. We also supplemented the Reuter expert topics with nonexpert topics obtained independently from three undergraduate students. Our objective was to increase the variety of gold standard topics, including some less specialized terms, synonyms, and semantically related words. For instance, a Reuter expert topic may consist of only “crude” but the nonexpert human subject could add more variety with topics such as “oil” or “energy.” This is important for a realistic evaluation of quality.

For the websites documents, the gold standard topics were assigned by the author due to logistical constraints and correspond to the categories listed previously, namely, transportation, technology, energy, pharmaceutical, food, vegetarianism/animal rights, sports, climate change, and universities. When it was thought that some of these categories could be more precise, more keywords corresponding to the more specific topics were added. For instance, the transportation topic was broken down into airline, train, and bus while the energy topic was further specified with keywords such as oil, wind, and solar. Hence, if a document is from an airline company website, its gold standard topics would be transportation and airline. Although the assignment of gold standard topics by the author for the web documents can be seen as a bias that may increase quality, we argue that the impact is minor for three reasons. First, to avoid preferential selection of gold standard topics, the web documents gold standard was created before observing topics generated by ReAD. This creates an “air gap” that makes the gold standard more independent of the author's potential biases. Second, the web document dataset was used as a verification of the results obtained with Reuter's set, for which all gold standard topics were obtained completely independently. Hence, if there were any biases in the preparation of the gold standards topics that resulted in better topics quality or different effects, one could always fall back onto the Reuter results as more valid. Lastly, even if there were some biases, it would have been very difficult to consciously or unconsciously predict the intricate behavior of ReAD and prepare the gold standard in such a way that the observed effects of STM and decay match anticipations and those of the Reuter set. That said, it will be important to reproduce our results with other datasets in future work as well as use independently constructed gold standards for evaluation.

Precision, recall, F1, accuracy, mutual information, and entropy are commonly used quality indicators for topics identification [29, 30]. They all compute quality based on matches between algorithmic topics and gold standard topics. However, in conducting previous work we found that these approaches are subject to anomalies when dealing with a varying and large number of topics, as is the case with ReAD. For instance, two documents discussing “an enterprise attempting to take over another one” might end up being assigned different topics such as “company ownership bid” and “corporate acquisition.” A typical case where this can happen is when different words are present in two documents discussing the same themes, which would result in the activation of different items. The different topics are not a problem as long as each set of topics conveys the correct idea about document content. However, automated topics quality evaluation methods fail to capture such semantic relationships. Furthermore, as the number of different topics grows, existing quality assessment approaches become distorted and do not provide a clear picture of the true quality of topics. The cause is an increasing spread of matches in the confusion matrix used to compute quality. This is in fact a problem not only for ReAD but also for any TI method for which topics do not come from a predefined list.

Because of the above issues with existing quality evaluation methods, we have elected to measure topics quality by a direct count of string matches between the topics generated by ReAD and those present in the gold standard. The advantage of counting string matches is that we obtain a value that directly and unambiguously states how well topics generated by the computational model correspond to those in the gold standard list.

Topics generated by ReAD are ordered by decreasing activation level because a higher activation is deemed to indicate a higher confidence that the topic is correct. The gold standard topics have no predefined order since they are all taken to be equally correct. One would hope to find the best topics among the first few in the ReAD ordered list. For this purpose, matches are counted separately for the top five and the top ten most activated topics, with the expectation that a larger proportion of matches with the gold standard would happen in the top five or ten. Topics beyond 10 are judged too far down the list to have been appropriately identified as topics. Five and ten are arbitrary values, but a fixed number of topics were necessary to perform quality evaluation, and these numbers fell within a range that seemed intuitively acceptable.

We now illustrate how the string match counting procedure works. For example, if ReAD outputs the following ordered topics for a document (top 10):

 
bank, company, rates, dollar, Canada, oil, sand, Alberta, exploitation, financing

while the gold standard topics for the same document are (without order)

 
dollar, price, corporation, oil, business, bank

then the match count for the first 5 topics would be 2 for “bank” and “dollar.” For the top 10 topics there is an additional match with “oil” and as such our quality evaluation procedure would report only one match.

For each pair decay rate and STM capacity, we sum all matches for all documents. So the quality we report is the total number of matches found between ReAD topics and human gold standard topics over all documents, one for the top 5 and the other for the top 10 topics. Also, we computed two separate upper quality baselines at decay = 0, one for the top 5 and the other for the top 10 topics.

## 4. Results and Discussion

**Figure 1:**
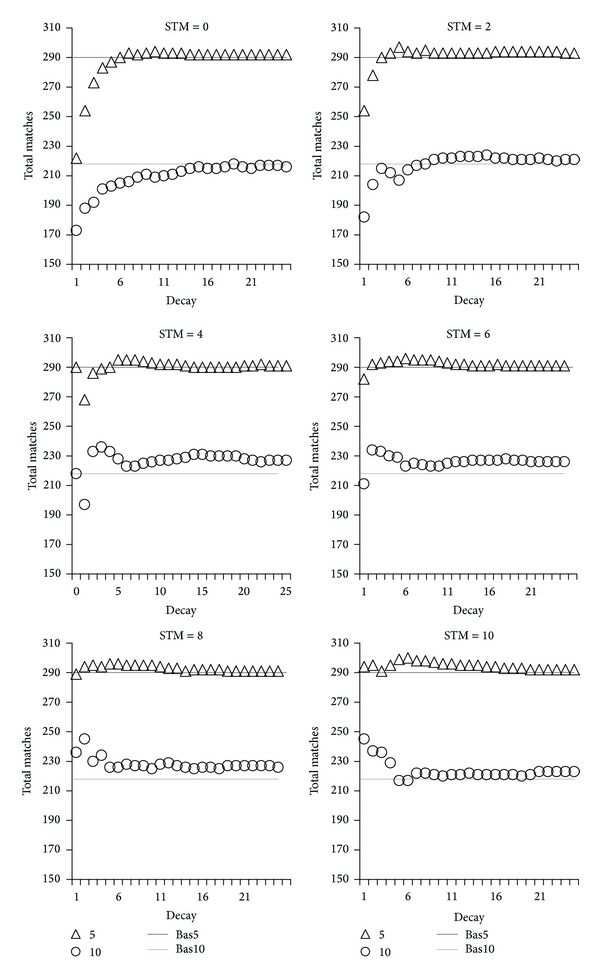
Effects of decay and STM capacity on topics quality for Reuter documents. Topics quality is expressed as total match counts (*y*-axis) with respect to decay rate (*x*-axis). Values on the *x*-axis correspond to *η*, the decay rate: a low *x*-axis value indicates rapid decay. Topics quality variations are shown at different STM capacity from 0 to 10, and for the first 5 (triangles) and next 5 (circles) most activated items produced by ReAD. Bas5 and bas10 indicate the upper baseline quality achieved in a nondecaying version of ReAD, respectively, for the first 5 and next 5 topics.

**Figure 2:**
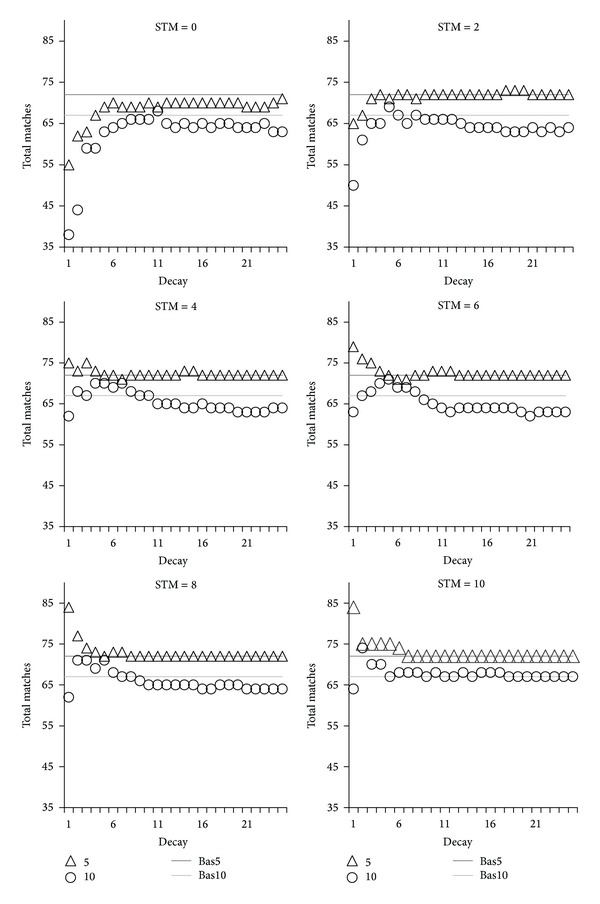
Effects of decay and STM capacity on topics quality for web sites documents. Topics quality is expressed as total match counts (*y*-axis) with respect to decay rate (*x*-axis). Values on the *x*-axis correspond to *η*, the decay rate: a low *x*-axis value indicates rapid decay. Topics quality variations are shown at different STM capacity from 0 to 10, and for the first 5 (triangles) and next 5 (circles) most activated items produced by ReAD. Bas5 and bas10 indicate the upper baseline quality achieved in a nondecaying version of ReAD, respectively, for the first 5 and next 5 topics.

An overview of the results is shown in Figure 1 for Reuter and Figure 2 for the web sites collection. Note that values on the *x*-axis correspond to the decay rate *η*, as per the decay formula in Algorithm 1. As such, a low *x*-axis value indicates rapid decay.

As expected, topics quality is negatively affected by rapid decay in the absence of STM. As decay slows, quality increases towards the baselines. These effects are clearly visible on the STM = 0 and STM = 2 graphs of Figures 1 and 2. In the case of STM = 2, there is insufficient STM to counteract decay, so the dip in quality still occurs. The low quality at rapid decay can be explained by items activation eroding too quickly, to the point that activation cannot accumulate as words are read. We have argued that activation is the computation commodity to estimate ideas frequency and therefore their importance.

Consequently when activation decays rapidly, it is not available or at an insufficient level to correctly identify ideas overlaps between words. On the contrary, for slower decay activation can accumulate, which explains why topics quality tends towards the baseline levels as we move to the right of the *x*-axis for STM = 0 and STM = 2.

We have claimed that decay is unavoidable in neural systems due to their bioelectric nature. If one assumes that decay is rapid, activation would not be an effective computational commodity without a mechanism to counteract it. We proposed that limited capacity STM serves this purpose by keeping the focus of attention on words and delaying the decay of their associated ideas. The next question we wanted to answer is whether STM would counteract the negative effect of decay on topics quality, or in other words does STM fix the dip in quality visible at rapid decay? We indeed observed the expected effect, evident in Figures 1 and 2 starting at STM = 4. Particularly interesting is that the dip in topics quality at rapid decay disappears as STM increases, to reach and even exceed the baselines.

Is this reversal of quality a positive side effect of decay, in the sense that it may filter out irrelevant items? The small increase in quality provided by STM at rapid decay seems to support this view. For confirmation, we visually inspected a small sample of documents to see if irrelevant items were indeed consistently ranked lower (i.e., filtered “out” from the top 10) with rapid decay compared to slower or no decay. The filtering effect of decay was inconclusive with some irrelevant items being ranked lower, but also in other cases rapid decay resulting in lower ranking of items that would make good topics and in higher ranking of irrelevant items. Although this analysis was imperfect and incomplete, being on a small scale and conducted by the main investigator, it did point out contradictory situations that do not support the proposal that decay* always* acts as a semantic filter. This is not a dramatic situation since the fundamental idea of including decay was biological plausibility, not to filter irrelevant items. The filtering is suggested as a potential positive side effect of otherwise detrimental decay. That being said, over the whole set of documents, rapid decay when coupled with STM did have a positive effect by overall ranking up more good topics than without decay, as evidenced by the small increase of quality. This is an interesting and unexpected effect.

Hence, given sufficient STM, quality that would otherwise be low due to rapid decay in fact increases. The gain compared to the no decay control baselines is small, but exceeding it is not the point. The important point is that given apparent inherent limitations caused by decaying neural systems, quality can be restored to the baseline level of nondecaying systems given a fairly small amount of STM. Indeed, STM does not need to have a large capacity: the reversal from low to high quality in our experiments is achieved in the general range of STM capacity humans are believed to possess.

For slow decay, STM does not matter. The role of STM appears to be of “stabilizing” quality under rapid decay conditions. This means that, thanks to STM, topics quality becomes independent of decay, so that even if decay is rapid or if it varies, the effectiveness of cognitive processes stays relatively constant and comparable to when there is no decay. This may be important for biological neural systems to maintain cognitive abilities irrespective of internal or external conditions that mainly affect decay speed.

Our experimental results put in evidence the importance of STM in the process of recognizing the main themes of natural language within a decaying neural system, thus validating the ReAD model. STM keeps words “meanings” active within a certain context as more words are read and other meanings fade away. Without STM, rapid decay causes the activation of relevant ideas to decrease and topics quality goes down. On the other hand, the presence of STM protects relevant ideas from immediate decay and quality increases. STM thus acts as a context preserver, allowing other words present in the neighborhood of a given word in the text to further activate shared ideas without decay taking place, while other ideas decay. In the end, only those ideas that were periodically and incrementally activated are ranked high. These represent the recurring themes in the text. Basically, ReAD exploits the following principle: STM capacity determines the neighborhood of a word in which shared ideas tend to occur. This is similar to existing corpus-based TI that treats cooccurrence of words as a sign of semantic relatedness, except that in our case relatedness is established by activating LTM ideas.

A related point is that STM may not only act as a neighborhood context preserver but also as stated when we described the ReAD model, for any recently fixated word. When we implemented ReAD, we only considered sequential words, but actual reading by humans involves more complex eyes movements. It would be interesting to implement such movements and observe any potential effect they may bring.

Our results also confirm the additional expectation that a larger proportion of good topics happen in the top five topics compared to the next five.

ReAD finds topics in a single document with no learning from linguistic data. Our aim when investigating single document TI was to focus our study on knowledge exploitation rather than acquisition. Instead of learning topics, our approach demonstrates how a mechanism such as LTM activation-decay with STM can generate topics. One may point out that we avoided the hard and interesting task of learning by simply using existing knowledge that was assembled at great effort by humans. One could also add that we replaced unstructured linguistic data with structured information in WordNet. We reiterate the following important points. First, our goal was to explore how the brain may implement the computation of ideas frequency as a means to identify topics. This required a way to simulate LTM ideas. WordNet was selected only in an attempt to emulate information stored in a person's brain. We do not dismiss that this information needs to be learned at some point. Second, we minimized the advantages potentially gained from structured WordNet entries by using individual words from all definitions as surrogates for brain ideas, without linguistic processing and without exploiting WordNet semantic relationships.

A final observation we would like to discuss is how our implementation of ReAD with a dictionary introduces noise that may not be present in a real brain, as alluded to in the experimental methodology section. This noise often takes the form of high frequency, “abstract” items from WordNet, masquerading as relevant items. These are words like “class,” “year,” “organization,” and so forth. This situation motivated the removal of so-called undesirable words from the list of items, but despite attempts to both manually and automatically remove them, there were always more appearing. For instance, we have tried tfidf and inversed entropy weighting without success. We applied these techniques “intradocument,” that is looking at a single document and collecting frequencies of items only for that document. Of note is that these techniques would normally call upon looking at the whole documents set, which we could not allow given that our aim is to process one document at a time in complete isolation of the others. One possible cause for the presence of these undesirable items is the wording of definitions that are often stated as X, is a Y where Y is a parent concept (e.g., “a cat is an animal”). Additionally, the WordNet information included hypernyms themselves containing items of a general or abstract nature. When such items are frequently appearing in WordNet definitions, they can produce topics that are too abstract to be useful in clearly conveying text topics. More work is required to resolve this aspect of the ReAD implementation, but in the end this seems to be an issue with implementation and not one with the ReAD model itself.

## 5. Conclusion

This paper focused on the following problems: what mental mechanisms are involved in identifying topics in natural language text? How do mental processes to identify topics exploit a person's existing knowledge? And, finally, what is the nature of the knowledge required to identify topics? We investigated these questions with experiments conducted with an implementation of the ReAD model. ReAD aims at modeling how humans identify topics when they read. Overall, the ReAD process consists of the neural activation and decay of information in LTM, as well as limited capacity STM that specifies the focus of attention on words and controls when decay is initiated. ReAD was motivated by the search for fundamental and innate mental mechanisms involved in topics identification and more precisely by the search for brain computational commodities to count frequently recurring ideas as a person reads, where the most frequent ideas are deemed to be the most important and thus identified as topics. Our aim was* not* to improve the efficiency and effectiveness of existing topics identification methods, and as such our experiments did not offer a comparison between ReAD and existing TI methods. We rather focused on verifying the effects of STM capacity and decay rate on the quality of topics produced by ReAD with the aim of determining if decaying activation of LTM ideas can serve as mental computational commodity in identifying topics.

The contribution of this paper was to show how activation and decay of loosely organized LTM ideas coupled with limited capacity STM interact to compute the topics of a text. Our work specifically demonstrated that activation and decay, provided adequate but limited STM capacity, serve as useful computational commodities to mentally estimate what ideas are the most important in natural language. We also confirmed that decay has adverse effects on cognitive processing, but that STM causes a reversal of the situation, going from worse quality to best for rapid decay. Our results suggest that fairly simple mechanisms as well as knowledge without formal structure are sufficient to identify topics. Although TI is a basic form of language understanding it is useful to guide further syntactic and semantic processing. These are interesting results that provide new insights into how basic semantic information may be derived by the brain, which could prove useful to develop new artificial intelligence systems and to better understand natural intelligence.

This work is only an exploratory first step that will require reproduction with refined evaluation methods and a variety of text datasets. In future work, we furthermore plan to investigate other factors that may influence topics quality, such as querying compound terms instead of individual words and exploiting basic linguistic preprocessing like part-of-speech tagging. We also plan to look into mechanisms to automatically eliminate undesirable items and compare various sources of LTM information surrogates.

## Figures and Tables

**Algorithm 1:**
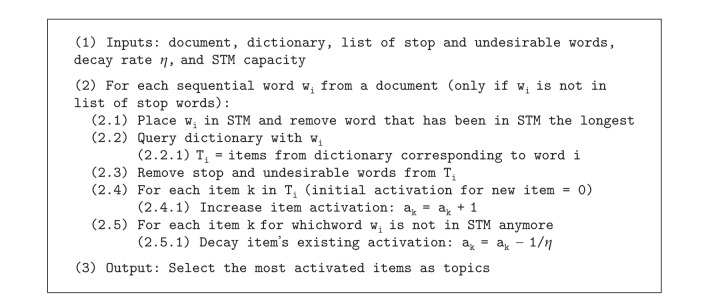
ReAD algorithm.
